# Research Methodology: Choices, Logistics, and Challenges

**DOI:** 10.1155/2014/780520

**Published:** 2014-04-14

**Authors:** Ian D. Coulter, George Lewith, Raheleh Khorsan, Ray Kirk, Brian Mittman

**Affiliations:** ^1^Rand Corporation, Santa Monica, CA 9040, USA; ^2^Primary Care and Population Sciences, University of Southampton, Southampton SO16, UK; ^3^Military Medical Research Program, Samueli Institute, Corona del Mar, CA 92625, USA; ^4^Department of Planning, Policy, and Design, University of California, Irvine, CA 92617, USA; ^5^School of Health Sciences, University of Canterbury, Christchurch 8140, New Zealand; ^6^Department of Veterans Affairs, VA Center for Implementation Practice and Research Support, Sepulveda, Greater Los Angeles, CA 91343, USA

Many complex issues are central to the ongoing debate about health care and health care delivery system reform in the United States (US) and worldwide. Academic medical centers, vulnerable populations, rural health, and hospitals represent but a few aspects of the fragmentation of the current health care delivery system. Although research offers considerable potential for generating insights into these issues, the challenge of developing and applying effective research methodology to study integrated health care delivery systems raises complex issues. In recent years, the roles, benefits, and challenges to appropriate use of research methods in the basic and applied social and clinical sciences have been debated extensively. These debates continue today.

The field of complementary and alternative medicine and integrative health care (CAM/IHC) is contributing exciting new developments in this ever challenging field. Over the past two decades in United States, CAM/IHC has experienced rapid growth in acceptance and use in the general population [1], with continued expansion into settings such as community hospitals, the US Department of Defense health care system [2], and the Veterans Health Administration [3]. Growth has also occurred in the body of research in CAM internationally [4], and CAM services are now being provided by new and innovative approaches such as health information technologies. Each of these provides new and exciting possibilities for CAM/IHC but equally each also provides new challenges. The collection of articles in this volume explores in various ways several of these challenges from the perspective of CAM/IHC research.

*Expanding Research Methodology*. Evidence-based practice (EBP) requires that decisions about health care are based on the best available, current, valid, and relevant research evidence. Major challenges to EBP include patient demographic trends, technological developments that increase the cost of health care, limitations in health care access and information, and lack of adequate resources and efficient methods to assess practices and produce required evidence. These challenges reduce the effectiveness, safety, and efficacy of CAM/IHC overall. Clinicians and researchers in CAM/IHC need greater opportunities to achieve improvements in individual and population health by increasing the production and application of research knowledge and the resulting clinical advances to enable more effective health care delivery.

Although worldwide growth in research funding that can support EBP in CAM/ICH is modest in real terms (e.g., at NIH it represents about 0.4% of the total research funding), recent growth has been considerable in the US and in Asia [5]. A study on the evolution of CAM in the US over the last 20 years by Jonas et al. [6] relying on literature indexes such as PubMed and MEDLINE showed that the terms alternative therapies or alternative medicine received 215, 502 citations in 2012, representing roughly a 70% increase in usage since 2004. The terms complementary therapies or complementary medicine received 185,819 citations, an increase of almost 60% in frequency of usage. The terms CAM or complementary and alternative medicine received 31,873 citations, a 122% increase in frequency of usage, while the term integrative medicine received 6,330 citations, a 310% increase in frequency of usage [6].

This growth is the result of several streams of activity. One stream is growth in the application of traditional research methods used in other areas (e.g., drug and clinical research, health services research, systematic reviews, and program evaluation) to CAM/IHC. Applications of methods from the fields of clinical research and health services research in particular have benefited from insightful, comprehensive debates and discussions of research methods and their appropriate use in CAM/IHC. Several of the articles included in this special issue illustrate this approach. A second stream of research is dealing with the unique challenges of CAM/IHC that demand innovative and adapted research methods. This stream is based on recognition that the biggest challenge for CAM/IHC research is development or adaptation of appropriate models of research that acknowledge therapeutic uniqueness while at the same time assuring that CAM/IHC research maintains standards of rigorous, valid science.

*Complexity of the Phenomena*. The first challenge for research methodology in CAM/ICH is the growing recognition that CAM/IHC practice often involves complex combination of novel interventions that include mind and body practices, holistic therapies, and others. Critics argue that the reductionist placebo controlled randomized control trial (RCT) model that works effectively for determining efficacy for most pharmaceutical or placebo trial RCTs may not be the most appropriate for determining effectiveness in clinical practice for either CAM/IHC or many of the interventions used in primary care, including health promotion practices [7]. Therefore the reductionist methodology inherent in efficacy studies, and in particular in RCTs, may not be appropriate to study the outcomes for much of CAM/IHC, such as Traditional Korean Medicine (TKM) or other complex non-CAM/IHC interventions—especially those addressing comorbidities [8, 9]. In fact it can be argued that reductionist methodology may disrupt the very phenomenon, the whole system, that the research is attempting to capture and evaluate (i.e., the whole system in its naturalistic environment). Key issues that surround selection of the most appropriate methodology to evaluate complex interventions are well described in the Kings Fund report [10] on IHC and also in the UK Medical Research Council (MRC) [11, 12] guidelines for evaluating complex interventions—guidelines which have been largely applied to the complexity of conventional primary care and care for patients with substantial comorbidity. These reports offer several potential solutions to the challenges inherent in studying CAM/IHC [11, 12].

*(1) Mixed Methods*. Two articles in this volume address the role of mixed methods. One analyzes the use of mixed methods approaches in CAM research. They reviewed publications in 10 major CAM journals in 2012 and found that 4% of papers (95 out of 2349) reported mixed methods studies, 80 of which met criteria for applying a quality appraisal tool. Quantitative components were generally of higher quality than qualitative components; when quantitative components involved RCTs they were of particularly high quality. Most strikingly, none of the 80 mixed methods studies addressed the philosophical tensions inherent in mixing qualitative and quantitative methods and none used an ethnographic approach (a core method within qualitative research) to explore the details of the interventions employed. This study conclude that the quality of mixed methods research in CAM can be enhanced by addressing these philosophical tensions, by improving reporting of analytic methods and reflexivity (in qualitative components), and through improved sampling and recruitment-related procedures and their reporting.

The second study used a mixed methods strategy (a combination of qualitative and quantitative approaches) to study the implementation of chiropractic programs in the U.S. Department of Veteran Affairs: this approach is discussed with a focus on the practical challenges encountered when using this mixed methods approach. They conclude with a series of specific recommendations, stating that “Analysis of qualitative observational data in studies combining deductive and inductive aims should be guided by pre-specified, model-based hypotheses and detailed analysis plans developed at the outset of the study. Unfortunately, while quantitative analysis methods are well-established and accepted, methods for analysis of qualitative data are subject to variability and lack of consensus. Analyses of qualitative data are too often informal, ad-hoc and emergent, with the possibility of low reliability and validity. These threats can be countered through the use of formal table approaches, in which key variables relevant to each hypothesis are listed in tables and manipulated in a blinded fashion, using qualitative pattern-identification and non-parametric quantitative techniques.”

*(2) Whole System Research*. A second response to the challenges of complexity in CAM/ICH research, usually combined with the use of mixed methods, adopts methods that avoid the reductionism thought to undermine much of RCT CAM related research and that permit evaluation of the whole system (the totality of the health encounter or the social system in which it is embedded). One powerful model for this is program evaluation. This special issue includes two examples using different models derived from program evaluation. The study of a community based integrative primary care practice uses program theory-driven science as an evaluation framework. Their main focus was on process evaluation (what actually happens in the real world) and adapted two widely used approaches: practice theory and fidelity evaluation; the practice theory component uses a logic model-based approach to evaluation. Other studies in this special issue utilize a whole system analytical approach such as that used by Donabedian's quality of care evaluation model. These studies show that whole systems evaluation can begin to understand effectiveness and complexity but requires a mixed methods approach to do so.

*(3) Comparative Effectiveness Trials/Pragmatic Trials*. The third, and more recent, response has increased interest in comparative effectiveness research (CER) in the US. The United Kingdom (UK) labels these approaches “pragmatic trials”; within the UK the* National Institute for Health Research* (NIHR) has supported this type of research as an important scientific foundation for clinical decision making. NIHR-funded CER is always associated with health economic evaluations and therefore allows for thoughtful decisions about the implementation of a particular treatment. In the US, the* Institute of Medicine* (IOM) defines CER as “the comparison of effectiveness of interventions among patients in a typical patient care setting with decisions tailored to individual need” [13]. In many ways, the move to CER and more pragmatic study designs should be beneficial for evaluating CAM interventions and is certainly at the core of primary care research and clinical decision making. Pragmatic trials can allow therapists to apply the treatments they choose providing they have model validity and they are closer to whole systems research than traditional placebo controlled RCTs. CER allows for variability in the way individuals are treated in trials and therefore comes closer to “personalized” medicine, another similarity with CAM.

The dilemma may be choosing between internal validity within a placebo controlled RCT and clinical relevance or generalizability within a pragmatic trial or CER study. As with conventional efficacy RCTs, CER establishes effectiveness but generally does not reveal the specific aspects of the intervention creating the effect. Mixed methods with a qualitative process evaluation component overlaid onto a pragmatic RCT helps to understand the why and how aspects of the health encounter as well as the issues around why people attend.

*(4) The Health Encounter, Context, Placebo, and Nonspecific Effects*. All clinical medicine, particularly within the community, represents a complex intervention and this is the case for both CAM and conventional medicine. Work within primary care clearly demonstrates that the encounter or consultation has a very substantial contextual effect which is dependent on many of the subtleties that exist within the doctor/patient relationship. This is a vital component of clinical practice and is not simply verbal but also nonverbal.

As work in the field of placebo effects has advanced, there has been recognition that much of the effect of CAM therapy that has been attributed to placebo is in fact a real effect [14–16]. But the proportions of CAM outcomes attributable to the therapy, placebo, and the context remain unclear. More sophisticated methods and studies are needed to determine what portion is attributable to each factor.

Two of the studies in this series begin to look at aspects of this issue. The first study reanalyzes data on homeopathy derived from cohort studies (real-world studies) to determine effectiveness. They present a rigorous statistical method for dealing with the problem of regression to the mean. Their work shows that changes in quality of life after treatment by a homeopath are small but cannot be explained by regression to the mean (RTM) alone.

The second study attempts to develop instruments to determine what nonspecific events are identified by patient centred measures. They present the methodology of an ongoing instrument development project that is “creating brief, precise, patient-reported measures of nonspecific factors that influence healing”.

How do we understand and measure the context of an encounter? [17] This issue is key to our understanding of CAM/IHC as recent research in homeopathy would suggest [18, 19]. Encounters are defined as what patients experience between when they arrive at a CAM practice site and when they leave. The encounter can be divided into two parts: (a) the experience of the main treatment intervention (e.g., what the provider does therapeutically to the patient during the visit) and (b) all other experiences before, during, and after the intervention itself. Contextual effects play two critical roles in assessing the efficacy and effectiveness of CAM interventions. First, contextual effects are likely to mediate how well a treatment works and may also contribute to outcomes directly. We refer to the former as context-as-mediator and the latter as context-as-intervention. In a classic RCT, investigators typically want to control for context-as-mediator effects and measure context-as-intervention effects to disentangle what portion of the results are due to the intervention and what are due to the context. In CER, investigators are less concerned about controlling for context-as-mediator effects, but many would like to understand what part of the encounter accounts for any positive results. In either case, investigators* must* know how to measure the components that create context. It is usually very useful to use the qualitative elements of a nested mixed methods approach to develop theoretical understanding of context within a particular illness/intervention and then to consider how best to understand the various contextual components using appropriate quantitative methodology.

This suggests that within CAM we must develop mixed methods approaches, involving qualitative and quantitative methods, that allow us to

1. understand what kinds of contextual factors patients are exposed to during CAM encounters;
2. determine how to measure such contextual factors reliably via observation and/or patient and provider recall or other means;
3. assess the degree to which contextual factors might vary within and across (a) CAM modalities, (b) practice sites, (c) providers, and (d) individual patient encounters;
4. assess and evaluate the impact of treatment or model.

*Conclusion*. Sufficient evidence exists now to suggest that the complexity of the health encounter in both CAM/IHC and conventional medicine, whatever therapy is utilized, requires a nonreductionist methodology if we are to advance beyond efficacy studies to studies of real-world effectiveness. A case can be made that effectiveness and cost effectiveness must be determined for an intervention to have any pragmatic usefulness in the world of patients and providers. While this is true in all health encounters it is particularly true for CAM/IHC interventions as they are new to most health care systems and may be expensive in therapist time. While this seems to be self-evident, what is not well-understood is the features of the encounter that most affect health outcomes. This highlights the need to determine what should be measured in the context of the therapeutic interaction and how it should be measured. It would also seem to be equally clear that we will require a mixed methods approach to develop these insights. While ethnographic observation techniques will be necessary to observe what happens in an encounter, it is not clear that observational ethnography can fully capture all of the nonverbal elements that may be very powerful within a consultation; we may need to videotape and record and score encounters using an approach such as the roter interaction analysis system (RIAS) [20, 21]. What is it about the clinical encounter that is truly powerful? Observation alone tells us that this is a multilayered and complex process but what are the important elements and how do we manipulate them? Ethnography, especially with appropriate video and audio recordings and scoring systems, will allow us to map the process but it will not allow us to attribute effectiveness to any specific element of this complex intervention.

We also need to elicit information from the players in the encounter, including providers, patients, staff, and auxiliaries. While it is important to know how they construct their individual reality and to know how they define their illness as well as their treatment and outcomes, we also need to measure these against more independent, objective measures. As noted by some of the studies in this special issue this methodology requires triangulation.

Achieving these goals requires that we locate or develop theories identifying what might or might not be important and then consider manipulating those aspects within clinical trials to see if we can understand their clinical impact. Ritual, as described and manipulated by Kaptchuk et al. [22] in patient with irritable bowel syndrome, is clearly important and sometimes therapy and ritual may be mistaken. The consultation itself plays a powerful effect, an effect that has as much impact on occasion as the therapy itself. However, we remain uncertain as to which elements of the health encounter are the most powerful and how we can apply new insights to achieve improved and more effective clinical care. These are all vital questions that need urgent answers and it is perhaps wise to consider whether our limited approach to mixed methods for complex interventions really goes far enough at present.

*Ian D. Coulter* *Ian D. Coulter* *George Lewith* *George Lewith* *Raheleh Khorsan* *Raheleh Khorsan* *Ray Kirk* *Ray Kirk* *Brian Mittman* *Brian Mittman*
